# Enhanced Intestinal Immune Response in Mice after Oral Administration of Korea Red Ginseng-Derived Polysaccharide

**DOI:** 10.3390/polym12102186

**Published:** 2020-09-24

**Authors:** Do Hwi Park, Byungcheol Han, Myoung-Sook Shin, Gwi Seo Hwang

**Affiliations:** 1 College of Korean Medicine, Gachon University, Seongnam-si, Gyeonggi-do 13120, Korea; parkdo@gc.gachon.ac.kr; 2 Efficacy & Safety Team, Korea Ginseng Corp., 30, Gajeong-ro, Yuseong-gu, Daejeon 34128, Korea; bchan@kgc.co.kr

**Keywords:** α-defensin-1, IgA, Korea red ginseng polysaccharide, Peyer’s patch

## Abstract

(1) Background: The immunostimulatory role of the polysaccharide fraction (KRG-P) of Korea red ginseng (KRG) was studied in cells. However, its immunomodulatory activity is unknown. Therefore, we investigated the chemical properties of KRG-P and its intestinal immune responses in vitro and in vivo. (2) Methods: KRG-P monosaccharide composition and molecular weight were determined using high-performance liquid and size-exclusion chromatography systems. Immunoglobulin A (IgA) and α-defensin-1 transcript levels were measured using a SYBR Green qRT-PCR; defensin-1, Granulocyte-macrophage colony-stimulating factor (GM-CSF), and IgA protein levels were determined using Western blotting and ELISA kits. (3) Results: The molecular weight of KRG-P was estimated to be 106 kDa, and it contained neutral sugar (74.3%), uronic acid (24.6%), and proteins (1%). In vitro studies of intestinal immunomodulatory activity of KRG-P indicated that GM-CSF and IgA levels increased in Peyer’s patch cells to higher levels than those obtained with KRG and induced bone marrow cell proliferation. In in vivo study, oral KRG-P administration to mice upregulated the expression of α-defensin-1 and IgA in the small intestinal tissue and that of secreted IgA in the feces. (4) Conclusions: KRG-P contributed to the modulation of intestinal immunity and maintenance of intestinal homeostasis against intestinal infection.

## 1. Introduction

The human body is exposed to foreign materials, such as pathogenic bacteria and viruses, but it maintains homeostasis through the immune system. The immune system is classified into primary (the thymus and bone marrow) and secondary lymphoid systems (the spleen, lymph nodes, and mucosal-associated lymphoid tissue (MALT)) [1,2]. MALT acts as a physical and immunological barrier against harmful substances and pathogenic bacteria [3]. Gut-associated lymphoid tissue has the largest MALT area and consists of Peyer’s patches, the lamina propria, and mesenteric lymph nodes, and Peyer’s patches are known as inductive sites that secrete IgA onto the mucosal surface [4]. B, T, and dendritic cells of Peyer’s patches are activated by antigens entering from the intestinal lumen, thereby secreting IgA that neutralizes the pathogens, and by producing cytokines, such as GM-CSF and Interleukine-6 (IL-6), which ultimately contribute to systemic immune activation [5]. In addition, Paneth cells present in the intestinal epithelium secrete an antimicrobial peptide called α-defensin-1 (Crytidin) after exposure to bacteria, lipopolysaccharide (LPS), lipid A, and muramyl dipeptide [6,7,8].

*Panax ginseng* C.A. Meyer has been widely used as a traditional medicine in East Asian countries such as Korea, China, and Japan. The pharmacological effects of *P. ginseng* against cancer [9], cardiovascular diseases [10,11], and diabetes [12], which are attributed to its active constituents such as ginsenosides, polyphenols, flavonoids, and polysaccharides, have been reported previously [13,14].

Red ginseng, which results from the heat treatment of white ginseng, has a higher content of ginsenoside and improved activities than those of white ginseng [15,16,17,18]. However, the pharmacological activities of red ginseng have not been attributed to ginsenosides. Recently, several studies reported that Korea red ginseng-derived polysaccharides showed immune-enhancing activities such as macrophage activation, cytokine production mediated by Peyer’s patches, and splenic lymphocyte proliferation in vitro and in vivo [19,20,21,22,23]. Nonetheless, there are few studies on the effects of polysaccharides from Korea red ginseng (KRG) on IgA production via Peyer’s patch activation and α-defensin expression for maintaining intestinal homeostasis in vivo. Therefore, in this study we extracted the polysaccharide fraction (KRG-P) from Korea red ginseng, analyzed its chemical properties, and investigated its possible role in intestinal immune activation. Our results indicated that KRG-P contributed to the modulation of intestinal immunity and the maintenance of intestinal homeostasis against intestinal infection.

## 2. Materials and Methods

### 2.1. Materials

The mouse antibodies against α-defensin-1 (ab122848) and β-actin (I-19) were purchased from Abcam (Cambridge, UK) and Santa Cruz Biotechnologies (Santa Cruz, CA, USA), respectively. Mouse GM-CSF Quantikine and IgA ELISA kits were provided by R&D Systems (Minneapolis, MN, USA) and Bethyl Laboratories (Montgomery, TX, USA), respectively. RPMI 1640 medium and penicillin/streptomycin were obtained from Gibco (Grand Island, NY, USA). Fetal Bovine Serum (FBS) ATCC 30-2020 was provided by ATCC (Manassas, VA, USA). Slide-A-Lyzer^®^ 20K dialysis Cassettes G2 were purchased from Thermo Scientific (Waltham, MA, USA). A Corning^®^ 100 µm Cell Strainer was obtained from Corning (Corning, NY, USA).

### 2.2. Preparation of the Polysaccharide Fraction from Red Ginseng

KRG extract, which has been recognized by the Ministry of Food and Drug Safety (formerly known as the Korea Food and Drug Administration) as a health functional food [24], was provided by Korea Ginseng Corporation (Seoul, Korea). KRG was first diluted ten times with purified water and then four times with ethanol, and the mixture was slowly stirred until precipitation of the polysaccharide fraction. The precipitate was collected by centrifugation (600× *g* for 20 min), dissolved in purified water, and then dialyzed with Slide-A-Lyzer 20K dialysis cassettes (molecular weight cut- off: 20,000 Da) to remove the ethanol and low-molecular substances and, finally, lyophilized.

### 2.3. Chemical Properties of KRG-P

The content of neutral sugars in the polysaccharide sample was determined based on the phenol–sulfuric acid method and using galactose as the standard [25]. The content of acidic sugar was quantified by m-hydroxybiphenyl using D-galacturonic acid as the standard substance [26]. The Bicinchoninic acid (BCA) method was used for protein quantification, using bovine albumin as the standard substance [27].

### 2.4. Determination of KRG-P Monosaccharide Composition and Molecular Weight

The KRG-P monosaccharide composition was determined by using a modified high-performance liquid chromatography (HPLC) method after pre-column derivatization with 1-phenyl-3-methyl-5-pyrazolone (PMP). The resulting PMP sugar derivatives were analyzed using the HPLC system equipped with an Acclaim^TM^ 120 C18 column (Thermo Scientific, Sunnyvale, CA, USA), as described in previous reports [28,29]. More details about the HPLC system and analytical conditions are described in Table 1. The molecular weight of KRG-P was determined by using high-performance size-exclusion chromatography with the Agilent 1260 Infinity liquid chromatography system (Agilent Technologies, Santa Clara, CA, USA) equipped with an Asahipak GS series (GS-520, GS-320, and GS-220) linked to the column (Showa Denko, Co. Ltd., Tokyo, Japan) and a refractive index detector (Agilent 1200 series). KRG-P was dissolved in distilled water (10 mg/mL, 10 μL) and analyzed using an isocratic buffer (50 mM ammonium formate buffer, pH 5.5). Molecular weights were determined from a calibration curve generated by using standard pullulans (P-5, 10, 20, 50, 100, 200, 400, and 800) (Showa Denko Co. Ltd. Minato-ku, Tokyo, Japan).

### 2.5. Animals

Female BALB/c mice (6–8 weeks old) were purchased from Orientbio (Seongnam, Korea) and housed at 23 ± 2 °C with 55% ± 10% humidity under a 12/12 h light/dark cycle, with free access to a standard laboratory diet and water. For in vivo assays, the mice were treated for 10 days with KRG or KRG-P at 5 or 50 mg/kg by using oral gavage feeding needles. Mice in the normal group were treated with sterilized distilled water. All animal experiments were performed in accordance with the instruction of the Ethics Comminttee for Use of Experimental Animals at Gachon University (2020-010).

### 2.6. GM-CSF and IgA Production Analyses in Peyer’s Patch Cells

BALB/c mice (7 weeks old, female) were sacrificed by cervical dislocation, and the small intestines were excised and placed on a sterilized paper. Peyer’s patches were dissected out using fine scissors from the wall of the small intestine and transferred to a Petri dish containing RPMI 1640 medium with penicillin/streptomycin. The tissues were minced by using a sterilized metal mesh (100 μm) to release immune cells. The cell suspension was filtered with a sterilized cell strainer and cultured on a cell culture plate at a concentration of 2.0 × 10^6^ cells/well (96-well plate), with RPMI 1640 medium containing 10% FBS, penicillin/streptomycin, and various concentrations of KRG or KRG-P at 37 °C in a humidified atmosphere (5% CO_2_, 95% air). After 5 days, the plate was centrifuged, and GM-CSF and IgA levels in the supernatants were analyzed using ELISA kits.

### 2.7. Bone Marrow Cell Proliferation Assay

The proliferating activity of Peyer’s patch-derived bone marrow cells was measured using the procedure reported previously [30,31,32]. In brief, mouse bone marrow cells were cultured with the supernatants collected from Peyer’s patch cells, and with KRG or KRG-P at a concentration of 125, 250, or 500 µg/mL. After 4 days, bone marrow cell proliferation was measured using 3-(4,5-dimethylthiazol-2-yl)-2,5-diphenyltetrazolium bromide (MTT)-based EZ-Cytox reagent. EZ-Cytox was added to each well, and the plates were incubated for an additional 2 h. The absorbance was then measured at 450 nm using the FilterMax F5 microplate reader (Molecular Devices, San Jose, CA, USA).

### 2.8. Quantitative RT-PCR

Mouse small intestinal tissues were minced with the TaKaRa BioMasher (Tokyo, Japan) and passed through a QIAshredder (Qiagen, Hilden, Germany) to reduce the viscosity of the solution. Total RNA from small intestinal tissues was isolated and purified using the RNeasy Mini kit (Qiagen, Hilden, Germany) and reverse-transcribed into cDNA by using the RevertAid First Strand cDNA Synthesis kit (Fermentas, Waltham, MA, USA), according to the manufacturers’ protocols. Quantitative RT-PCR was performed using a SYBR Green assay (Applied Biosystems, Foster City, CA, USA) and the indicated primers (Table 2). β-Tubulin was used as an internal gene control. cDNA amplification and analysis were performed by using a QuantStudio-3 real-time PCR system (Applied Biosystems).

### 2.9. Preparation of Tissue Lysate and Immuno-Blotting

To analyze protein expression in the mouse small intestine, the tissue was minced with cold radioimmunoprecipitation assay buffer (RIPA) buffer (Rockland, Limerick, PA, USA), supplemented with 1 mM dithiothteitol (DTT) (Merk, Darmstadt, Germany), and diluted with a phosphatase inhibitor cocktail 2 solution (Merk). After centrifugation, the amount of protein in each supernatant was quantified, mixed with an SDS sample buffer, and denatured for 5 min at 95 °C. Protein electrophoresis and transfer as well as membrane development were all performed as described previously [23,24]. Briefly, electrophoresis was performed using 12% Tris-glycine SDS-polyacrylamide gel, and the protein bands were transferred onto a polyvinylidene difluoride membrane, which was blocked for unspecific binding of proteins by incubation at room temperature with 5% skim milk. After three washes with a Tris-Buffered Saline buffer containing 0.1% Tween^®^ 20, the membrane was incubated with anti-α-defensin-1 or β-Actin antibodies for 3 h, followed by washes and incubation with a secondary antibody. The proteins were detected by using the FUSION Solo Vilber Lourmat system (Collégien, France) with an ECL solution. The intensities of the protein bands were quantified using the ImageJ program and captured images.

### 2.10. Statistical Analysis

The results were expressed as the mean ± standard deviation (SD) of duplicate or triplicate experiments. The results were statistically analyzed with Mann–Whitney using Prism 8 (GraphPad Software, San Diego, CA, USA).

## 3. Results and Discussion

### 3.1. Chemical Composition and Molecular Weight of KRG-P Extracted from KRG

**Figure 1:**
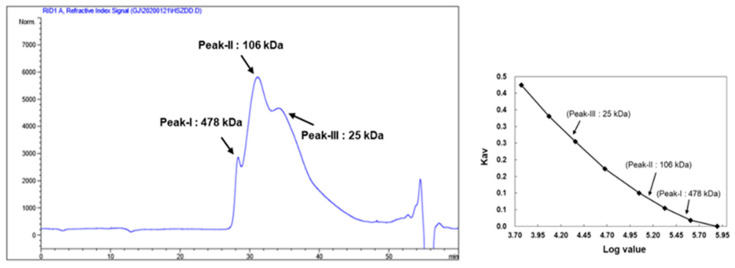
Molecular weight of KRG-P determined by high-performance size-exclusion chromatography, using Asahipak GS series GS-520 + GS530 + GS220 linked columns.

Polysaccharides from commercially available KRG were extracted; the yield of KRG-P was approximately 25% (*w*/*w* %). First, we analyzed the KRG-P chemical properties and monosaccharide composition (Table 3). KRG-P contained neutral sugars (74.3%), uronic acid (24.6%), and small traces of proteins (1.0%). The monosaccharide analysis suggested that KRG-P mainly consisted of glucose (60.5%), galacturonic acid (19.7%), galactose (11.0%), arabinose (6.8%), and rhamnose (1.9%). The KRG-P monosaccharide chromatogram is shown in Supplementary Figure S1. Next, we determined the KRG-P molecular weight by using HPLC equipped with a refractive index detector and Asahipak GS series (GS-520, GS-320, and GS-220) linked columns. As shown in Figure 1, KRG-P yielded three major peaks with molecular weights of 478, 106, and 25 kDa, respectively.

### 3.2. Effects of Intestinal Immunomodulatory Activity Mediated by Peyer’s Patch Cells

**Figure 2:**
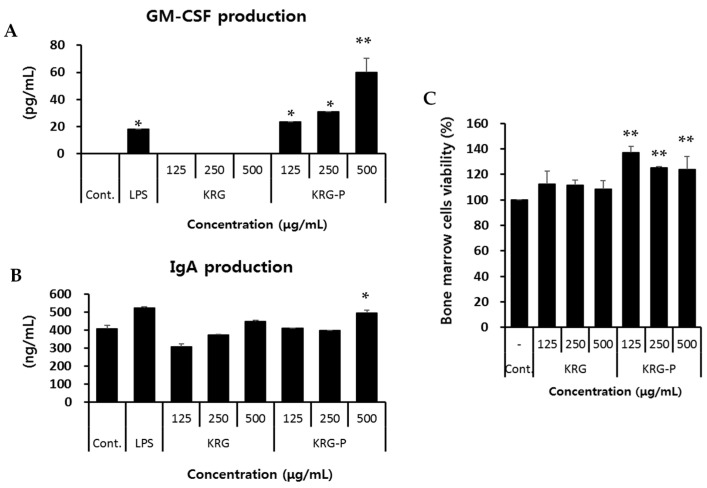
Effect of Korean red ginseng (KRG) and KRG-P on the immunomodulatory activity of intestinal Peyer’s patch cells. (**A**,**B**) In vitro GM-CSF and IgA production by Peyer’s patch cells was stimulated by KRG and KRG-P. Peyer’s patch cells (2.0 × 10^6^/mL) were treated with KRG and KRG-P in a 96-well plate for 4 days. The concentrations of GM-CSF and IgA in the Peyer’s patch cell supernatants were determined using ELISA kits. Lipopolysaccharide (LPS) was used as a positive control. Data are presented as the means ± SD of three independent experiments. (**C**) Bone marrow cell proliferation was induced by Peyer’s patches cells after treatment with KRG or KRG-P. Peyer’s patch cells (2.0 × 10^6^ cells/well) were treated with KRG or KRG-P in a 96-well plate for 4 days. The supernatants collected from these wells were added to the bone marrow cells (3.0 × 10^5^ cells/well) for 5 days. LPS was used as a positive control for bone marrow cell proliferation. Data are presented as the means ± SD of three independent experiments. * p < 0.05 vs. the normal group. ** p < 0.01 vs. the normal group.

Peyer’s patch-mediated intestinal immunity-enhancing activities of polysaccharides have been previously reported [21,30,31,32]. Thus, first we investigated the in vitro stimulatory activities of KRG and KRG-P on Peyer’s patch cells, which play a central role in the intestinal immune system. As shown in Figure 2A, KRG-P-treated Peyer’s patch cells strongly secreted GM-CSF in a concentration-dependent manner. However, KRG treatment did not affect GM-CSF production by Peyer’s patch cells. LPS was used as a positive control for GM-CSF production. IgA production by Peyer’s patch cells increased after treatment with 500 μg/mL KRG or KRG-P; nevertheless, only the last increase was statistically significant. Groups treated with lower concentrations of these compounds did not experience a change in IgA production (Figure 2B).

Next, we investigated bone marrow cell proliferation mediated by Peyer’s patch cells after treatment with KRG or KRG-P. The supernatants from Peyer’s patch cells incubated with KRG-P for 4 days significantly promoted ex vivo bone marrow cell proliferation (Figure 2C). The augmented bone marrow cell proliferation correlated with GM-CSF production by KRG-P, suggesting that KRG-P stimulated Peyer’s patch cells to produce cytokines, and consequently induced bone marrow cell proliferation. Collectively, these results indicated that KRG-P may stimulate intestinal immunomodulation.

### 3.3. Effects of KRG and KRG-P Oral Administration on Mouse IgA Production

**Figure 3:**
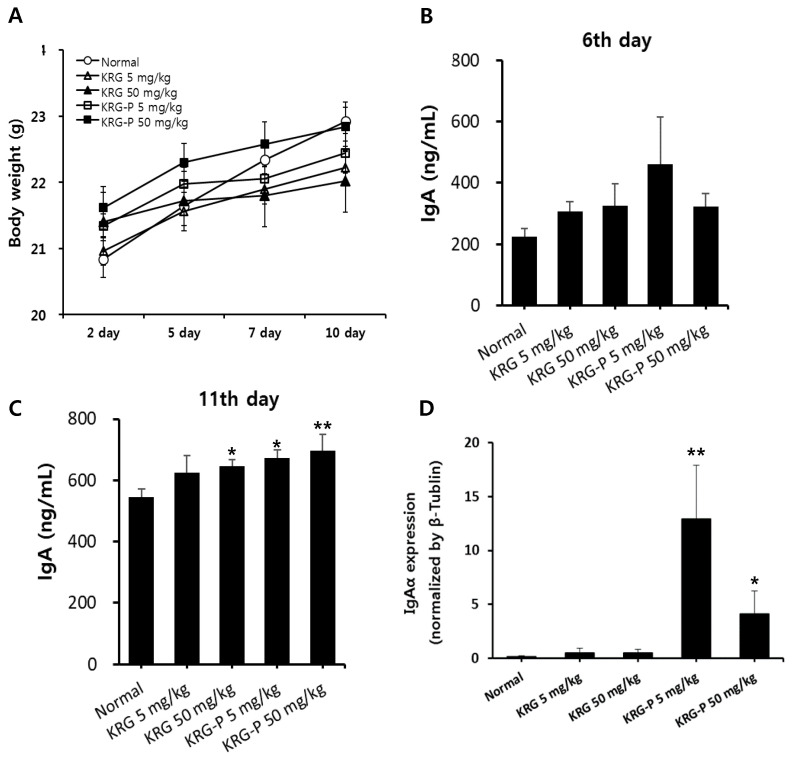
Effects of oral administration of KRG and KRG-P on mouse IgA production. (**A**) Five BALB/c mice per group were orally and daily administered with the indicated doses of KRG or KRG-P for 10 days. Bodyweight was estimated every two or three days. (**B**,**C**) Mouse fecal samples were collected on days 5 and 11 and analyzed for IgA expression by using mouse ELISA assays. (**D**) The mice were sacrificed at 11 days after oral treatment, and tissues from the jejunum region of the small intestine were then collected. Intestinal IgA mRNA levels were measured using qRT-PCR. Data are presented as the means ± SD of three independent experiments. * p < 0.05 vs. the normal group. ** p < 0.01 vs. the normal group.

Based on the in vitro results, we investigated the in vivo intestinal immunomodulation activity of KRG and KRG-P. According to our previous report [26], activation of innate immune cells, such as natural killer (NK) cells and macrophages, was confirmed after oral administration of natural polysaccharides at 5–50 mg/kg to mice. Therefore, in this experiment, KRG or KRG-P was orally and daily administered to BALB/c mice for 10 days at 5 and 50 mg/kg. During the oral administration of KRG or KRP-P, we measured the bodyweight of each mouse every 2–3 days. As shown in Figure 3A, the bodyweight of the normal group slightly increased, and that of the KRG or KRG-P group showed similar patterns. These results indicated that KRG or KRG-P treatment did not affect the bodyweight of mice (Figure 3A).

Next, to verify the modulating activity of KRG or KRG-P on the intestinal immune system, we measured IgA production in mouse feces on days 6 and 11 after oral administration. On the 6th day, IgA production increased in all treatment groups, but not significantly, compared with that in the normal group (Figure 3B). However, on the 11th day, secreted IgA levels significantly increased in the KRG-P-treated group in a dose-dependent manner, and in the KRG-treated group only at the concentration of 50 mg/kg (Figure 3C). In addition, to confirm IgA secretion in feces, we measured IgA mRNA expression levels in the jejunum region of the intestinal tissue. As shown in Figure 3D, the KRG-P-treated group (5 and 50 mg/kg) exhibited a significant increase in IgA mRNA levels. Collectively, oral administration of KRG-P augmented IgA production in feces and intestinal tissue; meanwhile, KRG administration produced a slight increase in IgA production. These results suggested that polysaccharides, rather than ginsenosides, of red ginseng may affect IgA production in mice.

### 3.4. Effects of KRG and KRG-P Oral Administration on Intestinal Mouse α-Defensin-1 Expression

**Figure 4:**
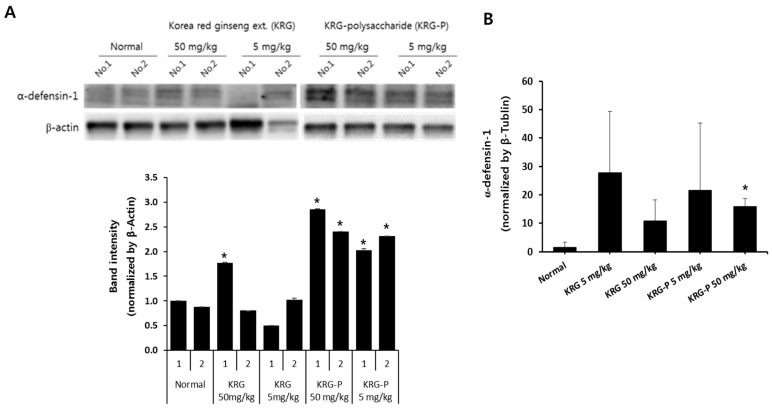
Effects of oral administration of KRG and KRG-P on α-defensin-1 expression in mouse intestinal tissues. BALB/c mice were orally administered the indicated doses of KRG or KRG-P daily for 10 days. (**A**) e α-defensin-1 protein levels were determined by immunoblotting with the specific antibody. Actin was used as a loading control. The bar chart displays the intensity of α-defensin-1 after normalizing with that of β-actin by using ImageJ software. (**B**) α-defensin-1 mRNA levels were determined by using qRT-PCR. An actin gene was used as a housekeeping gene for normalization. Data are presented as the means ± SD of three independent experiments. * p < 0.05 vs. the normal group.

Paneth cells, present in the intestinal epithelium, produce and secrete α-defensin, lysozyme, and proinflammatory mediators that play a role in maintaining intestinal homeostasis [6,7]. Therefore, we determined the protein levels of α-defensin-1, an antimicrobial and antiviral peptide, in the intestinal tissues of mice orally administered KRG or KRG-P. As shown in Figure 4A, α-defensin-1 protein expression in the jejunum markedly increased after oral administration of 5 and 50 mg/kg KRG-P compared with that in the control group, and after 50 mg/kg KRG administration, which showed a slight increase. KRG-P and KRG administration increased the mRNA expression of α-defensin-1 compared with the control group (Figure 4B). In most of the 50 mg/kg KRG-P-treated mice, the mRNA and protein levels of α-defensin-1 significantly increased (Figure 4B). Meanwhile, the observed increase in α-defensin-1 mRNA levels was not statistically significant in the group receiving 5 mg/kg compared with that in the normal group. These results suggested that oral administration of KRG-P may have better protective activity against intestinal infections than KRG.

## 4. Conclusions

The intestinal tract is exposed to foreign substances, such as food and pathogenic bacteria. Therefore, maintaining homeostasis in the intestine is particularly important for the defense mechanism of the body. Peyer’s patches, an important organ of the intestinal tract, contains specialized M cells in the epithelium that uptake antigens from the intestinal lumen. These antigens activate B-lymphocytes in the Peyer’s patch, which secrete IgA to prevent mucosal infections. In this study, we first confirmed that KRG-P stimulated Peyer’s patch cells to produce GM-CSF and IgA and induced bone marrow cell proliferation ex vivo (Figure 2). Recently, Kim et al. reported that α-amylase- and amyloglucosidase-treated polysaccharides (non-starch-like fraction) isolated from KRG induced the significant production of IL-6 and GM-CSF by Peyer’s patches [21]. Yu et al. reported that hot water-extracted crude polysaccharide fraction isolated from *Atractylodes lancea* DC showed bone marrow cell proliferation activity mediated by Peyer’s patches [31]. Furthermore, we showed that oral administration of KRG-P (5 and 50 mg/kg) increased IgA secretion in mouse feces on day 11 (Figure 3C); mRNA expression of IgA also increased in the 50 mg/kg-treated group (Figure 3D). These results suggested that polysaccharide fractions can stimulate Peyer’s patch immune cells and produce cytokines and IgA. IgA is mainly produced by Peyer’s patches and plays a central role in protection against pathogens and homeostatic regulation of the intestine. Thus, our results showed that KRG-P has intestinal immune stimulatory activity. Moreover, we showed that protein and mRNA expression levels of α-defensin-1 were significantly increased by oral administration of 50 mg/kg KRG-P (Figure 4). These results suggested that KRG-P possessed protection activity against intestinal infection and maintained homeostasis of the intestinal lumen through activation of intestinal immunity. Future studies should focus on the effects of KRG-P on the expression of intestinal tight junction proteins, such as E-cadherin, ZO-1, and occludin, that are related to the regulation of intestinal barrier permeability and changes of the microbiome.

## Figures and Tables

**Table 1:** HPLC analytical conditions to determine polysaccharide fraction (KRG-P) monosaccharide composition.

Apparatus	Liquid Chromatography LC-20AD (Shimadzu Corporation, Kyoto, Japan)
Detector	UV/VIS detector (Shimadzu Corporation, Kyoto, Japan) monitored at 245 nm
Column	Acclaim^TM^ 120 C18 (Thermo Scientific, Sunnyvale, CA, USA)
Column size	4.6 × 250 mm (5 μm particle size)
Column temperature	30 °C
Flow rate	1 mL/min, isocratic elution
Eluent	0.1 M sodium phosphate buffer (pH 6.7): Acetonitrile (82: 18)
Injection volume	10 μL
Integrator	Shimadzu data module (Shimadzu Corporation, Kyoto, Japan)

**Table 2:** Primer sequences used for quantitative RT-PCR.

Gene Name	Forward Primer	Reverse Primer
Mouse IgA	5′-TGAGCGCTGGAACAGTGGCG-3′	5′-TCAGGGCCAGCTCCTCCGAC-3′
Mouse α-defensin-1	TaqMan Mm02524428	
Mouse β-tubulin	5′-CTCCCAGGTTAAAGTCCTTCAGTA-3′	5′-GCAACATAAATACAGAGGTGGCTA-3′

**Table 3:** Monosaccharide composition of KRG-P isolated from KRG-P.

Chemical Property (%) ^1^	KRG-P
Neutral sugar	74.3 ± 0.03
Uronic acid	24.6 ± 0.02
Protein	1.0 ± 0.05
**Component sugar**	(Mole %)
Rhamnose	1.9 ± 0.2
Fucose	-
Arabinose	6.8 ± 0.1
Xylose	-
Mannose	-
Galactose	11.0 ± 0.0
Glucose	60.5 ± 0.0
Glucuronic acid	-
Galacturonic acid	19.7 ± 0.1

^1^ Percentage (%) against the dried crude polysaccharide.

## Supplementary Materials

The following are available online at https://www.mdpi.com/2073-4360/12/10/2186/s1, Figure S1: A HPLC chromatograms KRG-P monosaccharide analysis.
