# Supplementary material for: Enhanced Intestinal Immune Response in Mice after Oral Administration of Korea Red Ginseng-Derived Polysaccharide
Source: Polymers (Basel). 2020 Sep 24;12(10):2186. doi: 10.3390/polym12102186 (PMC7600159; doi:10.3390/polym12102186)
Supplement: Supplementary file 1 [file polymers-12-02186-s001.pdf]

Enhanced Intestinal Immune Response in Mice after Oral Administration of Korea Red Ginseng-Derived Polysaccharide

Do Hwi Park [1], Byungcheol Han [2], Myoung-Sook Shin [1,*] and Gwi Seo Hwang [1,*]

1 College of Korean Medicine, Gachon University, Seongnam-si, Gyeonggi-do 13120, Korea; parkdo@gc.gachon.ac.kr
2 Efficacy & Safety Team, Korea Ginseng Corp., 30, Gajeong-ro, Yuseong-gu, Daejeon 34128, Korea; bchan@kgc.co.kr
* Correspondence: ms.shin@gachon.ac.kr (M.-S.S.); seoul@gachon.ac.kr (G.S.H.); Tel.: +82-31-750-5423 (M.-S.S.); +82-31-750-5421 (G.S.H.)

Received: 18 August 2020; Accepted: 23 September 2020; Published: date

Supplementary material

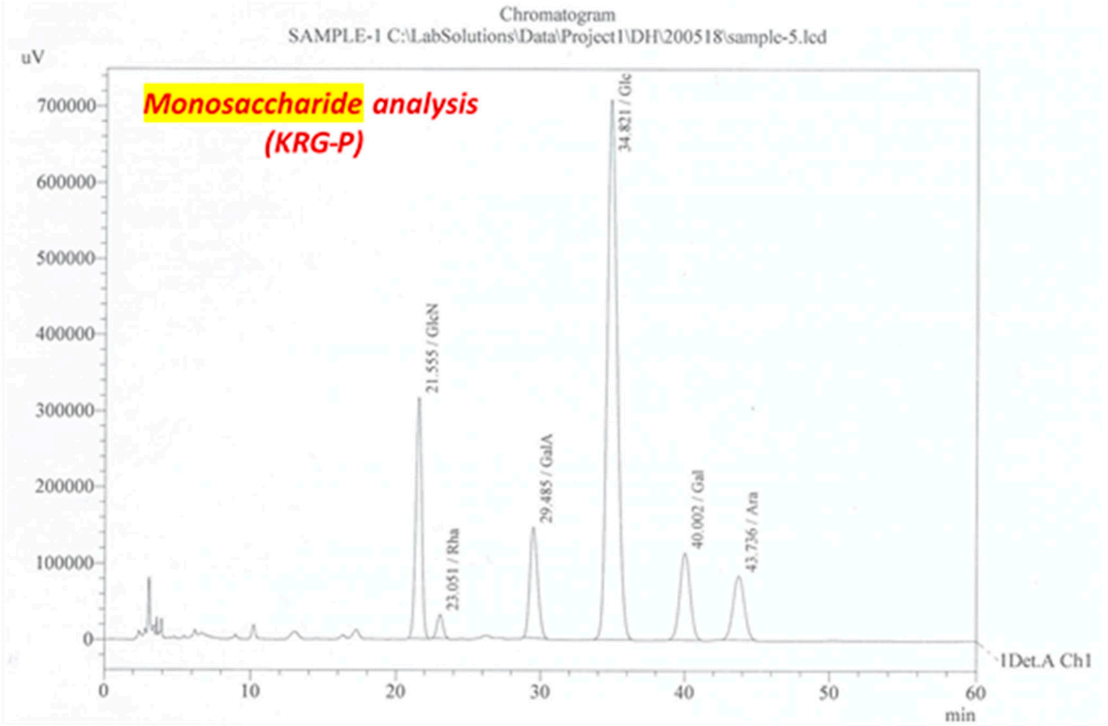

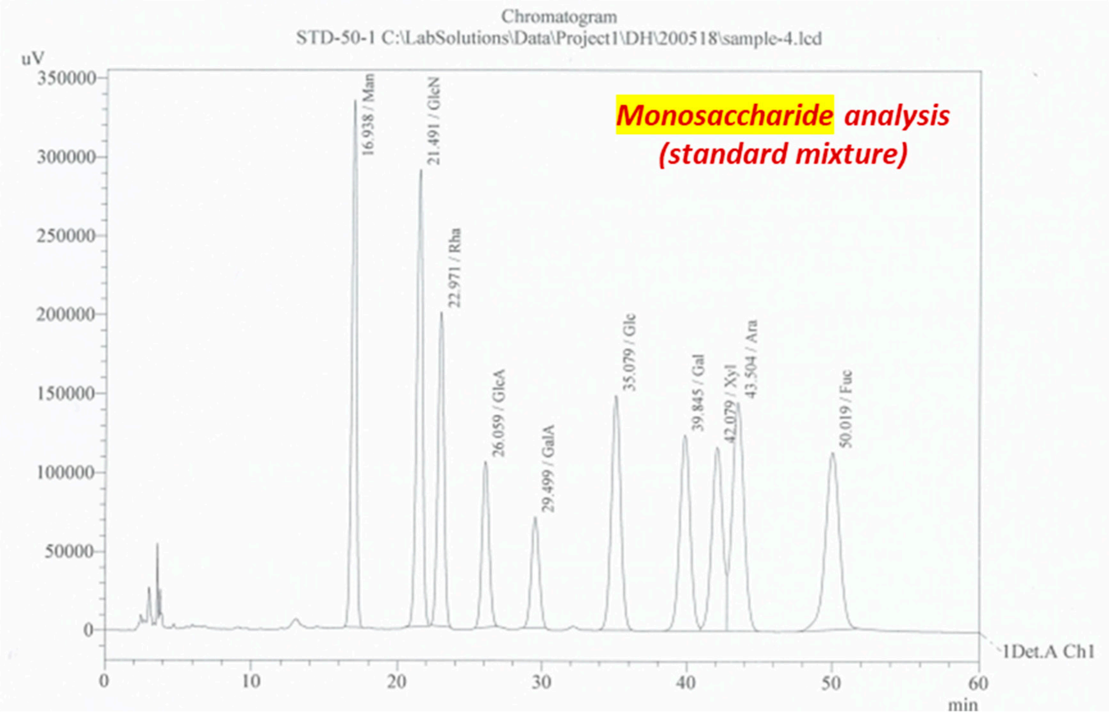

Figure S1. A HPLC chromatograms KRG-P monosaccharide analysis.
